# Predictive values of the SOFA score and procalcitonin for septic shock after percutaneous nephrolithotomy

**DOI:** 10.1007/s00240-022-01366-7

**Published:** 2022-10-10

**Authors:** Haifeng Hou, Jun Yang, Zhenhua Han, Xiaoyang Zhang, Xiaoying Tang, Tianming Chen

**Affiliations:** 1 Department of Urology, Jiangjin Central Hospital, No. 725, Jiangzhou Road, Dingshan Street, Jiangjin District, Chongqing, 402260 China; 2 Department of Critical Care Medicine, Jiangjin Central Hospital, No. 725, Jiangzhou Road, Dingshan Street, Jiangjin District, Chongqing, 402260 China

**Keywords:** Percutaneous nephrolithotomy, Sequential organ failure assessment score, Procalcitonin, Prediction

## Abstract

To investigate the value of combination of the Sequential Organ Failure Assessment (SOFA) score and procalcitonin (PCT) for prediction of septic shock after percutaneous nephrolithotomy (PCNL). A total of 1328 patients receiving PCNL for renal calculi were allocated into control group (without septic shock) and septic shock group, and related data were retrospectively collected. Univariate analysis was firstly performed, and the variables with two sided *P* < 0.10 were then included in *logistic* regression analysis to determine independent risk factors. Receiver operating characteristic (ROC) curve was utilized to evaluate the predictive values. Area under curve (AUC) was compared using *Z* test. Postoperative septic shock was developed in 61 patients (4.6%) and not developed in 1267 patients (95.3%). Multivariate analysis demonstrated that SOFA score (OR: 1.316, 95% CI 1.125–1.922), PCT (OR: 1.205, 95% CI 1.071–1.696) and operative time (OR: 1.108, 95% CI 1.032–1.441) were independent risk factors for septic shock with adjustment for sex, history of urolithiasis surgery, positive history of urine culture and history of PCNL. The ROC curves demonstrated that the AUCs of SOFA score and PCT for predicting septic shock after PCNL were 0.896 (95% CI 0.866–0.927) and 0.792 (95% CI 0.744–0.839), respectively. The AUC of their combination was 0.971 (95% CI 0.949–0.990), which was higher than those of individual predictions (vs 0.896, *Z* = 4.086, *P* < 0.001; vs 0.792, *Z* = 6.983, *P* < 0.001). Both the SOFA score and PCT could be applied in predicting septic shock after PCNL, and their combination could further elevate the diagnostic ability.

**Supplementary Information:**

The online version contains supplementary material available at 10.1007/s00240-022-01366-7.

## Introduction

At present, percutaneous nephrolithotomy (PCNL) is the most appropriate treatment in patients with renal calculi of > 2 cm [1]. As one of the most catastrophic complications after this procedure, sepsis remains a challenging health concern around the world with increasing incidence in spite of sterile preoperative prophylactic and urine antibiotics [2, 3]. Sepsis is characterized by a fatal organ dysfunction, which correlates with a dysregulated host response to infection [4]. As a subset of sepsis, septic shock can affect the cellular metabolism and cardiovascular system, leading to increased mortality. The incidence of septic shock among patients undergoing PCNL ranges from 0.3 to 9.3% [5–7], and its mortality is up to 66% in some investigations [8, 9]. Therefore, early prediction and treatment of septic shock is helpful in decreasing the mortality and improving the prognosis.

The Sequential Organ Failure Assessment (SOFA) score can be applied in grading sepsis and predicting the prognosis of patients [10]. Moreover, the SOFA score is related to septic shock after PCNL and have a high predictive value [11]. Procalcitonin (PCT) has been demonstrated to be a valuable laboratory indicator for early prediction of sepsis and differentiation of septic shock, severe sepsis and sepsis [12]. In addition, PCT is also shown to be a useful tool in monitoring the effectiveness of antibiotic therapy among sepsis patients [13, 14]. However, the value of combination of SOFA score and PCT for prediction of septic shock after PCNL is still not evaluated.

## Methods

### Study design and setting

This was a retrospective case–control study performed at Jiangjin Central Hospital. Related data of all included patients were collected through reviewing their medical records.

### Participants

A total of 1328 patients receiving PCNL for renal calculi between January 2016 and June 2021 were retrospectively recruited. Their inclusion criteria included ① possessing one symptom of urinary tract infection at least (urgency, dysuria, perineal pain, frequency, costovertebral tenderness or flank pain); ② greater than five leukocytes per high-power field in a centrifuged sediment before PCNL or leukocyturia or a positive nitrite dipstick test; ③ no serious cerebro- and cardiovascular diseases and hemorrhagic tendency; and ④ complete medical records. Their exclusion criteria included ① current treatment for hydronephrosis; ② a previous history of kidney transplantation; and ③ hemoor peritoneal dialysis, pregnancy or presence of polycystic kidney disease. This study was carried out according to the guidelines outlined by the Declaration of Helsinki, and the study protocol received the approval of the Ethics Committee of Jiangjin Central Hospital (JJ021014). All participants provided written informed consents.

### Variables

The variables analyzed in this study included demographic data, clinical data, operation data and characteristics of stone. The definition of the SOFA criteria was described by the Sepsis-3 Task Force, including platelet, oxygenation index, bilirubin, the vasoactive drugs support and mean arterial pressure, Glasgow coma score (GCS), and creatinine and urine output. The SOFA score was evaluated daily during hospitalization to determine whether septic shock had developed. The SOFA score in the 24 h after PCNL was taken as predictive value. The highest SOFA score was taken as documenting septic shock. PCT was also evaluated in the 24 h after PCNL, which was taken as predictive value.

### Grouping and diagnostic criteria

All participants were allocated to control group (without septic shock) and septic shock group. The diagnostic criteria of septic shock included ① the SOFA score ≥ 2 points; and ② a serum lactate level > 2 mmol/L and persistent hypotension requiring vasopressors to keep mean arterial pressure ≥ 65 mmHg in spite of adequate volume resuscitation.

### Statistical analysis

Processing and statistical analysis of all data were carried out with SPSS version 18.0 (SPSS Inc., USA). Student's *t* test was used to compare intergroup differences of continuous variables with normal distribution and Mann–Whitney *U* test was used to compare intergroup differences of those without normal distribution. Chi-square test was used to compare intergroup differences of categorical variables. The variables of two sided *P* < 0.10 were then included in logistic regression analysis to determine independent risk factors. The predictive values were evaluated using receiver operating characteristic (ROC) curve. *Z* test was used to compare the area under curve (AUC). Two sized probability values (*P*) < 0.05 were considered statistically significant.

## Results

### General data

These 1328 patients included 771 males and 557 females. The raw data were provided in Dataset 1 as supplementary material. Their average age was 52.96 ± 12.03 years, average body mass index (BMI) was 24.18 ± 2.82, and stone burden was 5.1 (2.3–9.7) cm^3^. Among them, 374 patients (28.2%) were complicated with hypertension and 145 (10.9%) patients with diabetes. Postoperative septic shock was developed in 61 patients (4.6%) and not developed in 1267 patients (95.3%).

### Univariate analysis

According to univariate analysis results (Table 1), the differences of sex, history of urolithiasis surgery, positive history of urine culture, SOFA score, PCT and operative time were statistically significant between septic shock group and control group (*P* < 0.05), and the differences of the rest variables were not statistically significant (*P* > 0.05). But the *P* value of history of PCNL was < 0.10.

**Table 1:** Univariate analysis results between septic shock group and control group

Variables	All patients (*N* = 1328)	Septic shock group (*n*1 = 61)	Control group (*n*2 = 1267)	*χ*^*2*^/*Z*/*t*	*P*
Male (*n*, %)	771(58.1%)	27(44.3%)	744(58.7%)	4.997	0.025
Age (years, mean ± SD)	52.96 ± 12.03	53.27 ± 11.82	52.95 ± 12.05	0.206	0.847
BMI (Kg/m^2^, mean ± SD)	24.18 ± 2.82	23.71 ± 3.38	24.20 ± 2.79	– 1.114	0.276
Comorbidities (*n*, %)					
Diabetes mellitus	145(10.9%)	6(9.8%)	139(11.0%)	0.077	0.781
Cardiovascular disease	38(2.9%)	2(3.3%)	36(2.8%)		0.692^a^
Hypertension	374(28.2%)	21(50.0%)	353(39.5%)	1.240	0.265
Functional solitary kidney	67(5.0%)	5(8.2%)	62(4.9%)		0.229^a^
Chronic kidney disease	99(7.5%)	8(13.1%)	91(7.2%)		0.126^a^
History of urolithiasis surgery (*n*, %)					
PCNL	270(20.3%)	18(29.5%)	252(19.9%)	3.294	0.071
Open surgery	221(16.6%)	14(23.0%)	207(16.3%)	1.835	0.176
EWL	173(13.0%)	5(8.2%)	168(13.3%)	1.317	0.251
URS	155(11.7%)	9(14.8%)	146(11.5%)	0.589	0.443
No	509(38.3%)	15(24.6%)	494(39.0%)	5.105	0.024
Preoperative drainage (*n*, %)					
Ureteral double-J stenting	179(13.5%)	11(18.0%)	168(13.3%)	1.137	0.286
Percutaneous nephrostomy	103(7.8%)	4(6.6%)	99(7.8%)		1.000^a^
Positive history of urine culture (*n*, %)	568(42.8%)	61(100.0%)	507(40.0%)		< 0.001^a^
Characteristics of stone					
Stone number [*n*, *M*, (*IQR*)]	4(4–4)	4(4–4)	4(4–4)	0.117	0.904
Stone burden [cm^3^, *M*, (*IQR*)]	5.1(2.3–9.7)	7.9(2.5–15.7)	5.0(2.3–9.6)	0.921	0.349
Stone localization (*n*, %)					
Upper calyx	317(23.9%)	18(29.5%)	299(23.6%)	1.118	0.290
Middle calyx	573(43.1%)	28(45.9%)	545(43.0%)	0.198	0.657
Lower calyx	447(33.7%)	17(27.9%)	430(33.9%)	0.960	0.327
Pelvis	114(8.6%)	7(11.5%)	107(8.4%)	0.681	0.409
Staghorn	136(10.2%)	10(16.4%)	126(9.9%)	2.633	0.105
Multiple	394(29.7%)	21(34.4)	373(29.4%)	0.694	0.405
SOFA score (mean ± *SD*)	2.40 ± 1.13	6.32 ± 3.18	2.21 ± 1.03	10.069	< 0.001
Antibiotic treatment time [*d*, *M*, (*IQR*)]	3(2–5)	4(3–5)	3(2–5)	1.146	0.305
PCT [ng/mL, mean ± SD]	10.83 ± 5.91	18.32 ± 9.13	10.47 ± 5.76	6.652	< 0.001
Number of punctures (*n*, %)					0.180^a^
≥ 3	95	7	88		
< 3	1233	54	1179		
Operative time (*n*, %)				11.634	0.001
≥ 90 min	323	26	297		
< 90 min	1005	35	970		
Fragmentation method (*n*, %)				0.082	0.775
Ureteroscope	936	42	894		
Nephroscope	392	19	373		

*BMI* body mass index, *SOFA* sequential organ failure assessment, *PCT* procalcitonin, *M* median, *IQR* inter quartile range, *PCNL* percutaneous nephrolithotomy, *SD* standard deviation

^a^Fisher's Exact Test

### Multivariate analysis

Multivariate analysis was carried out for sex, history of urolithiasis surgery, positive history of urine culture, SOFA score, PCT, operative time and history of PCNL using the binary logistic regression model. The analysis results (Table 2) demonstrated that SOFA score, PCT and operative time were independent risk factors for septic shock with adjustment for sex, history of urolithiasis surgery, positive history of urine culture and history of PCNL.

**Table 2:** Multivariate analysis results between septic shock group and control group

Variables	*β*	SE	Wald *χ*^*2*^	OR	95% CI	*P*
SOFA score	0.470	0.201	6.873	1.316	1.125–1.922	0.004
Operation time	0.156	0.070	4.408	1.108	1.032–1.441	0.036
PCT	0.207	0.072	5.901	1.205	1.071–1.696	0.013
Male	– 0.103	0.037	2.048	0.759	0.605–1.273	0.159
History of urolithiasis surgery	0.128	0.027	1.942	1.386	0.753–1.849	0.215
Positive history of urine culture	0.147	0.046	2.442	1.235	0.867–1.523	0.123
History of PCNL	0.078	0.009	1.249	1.351	0.746–1.834	0.278

*SE* Standard error, *OR* odds ratio, *CI* Confifidence interval, *SOFA* sequential organ failure assessment, *PCT* procalcitonin

## Predictive value

**Fig. 1:**
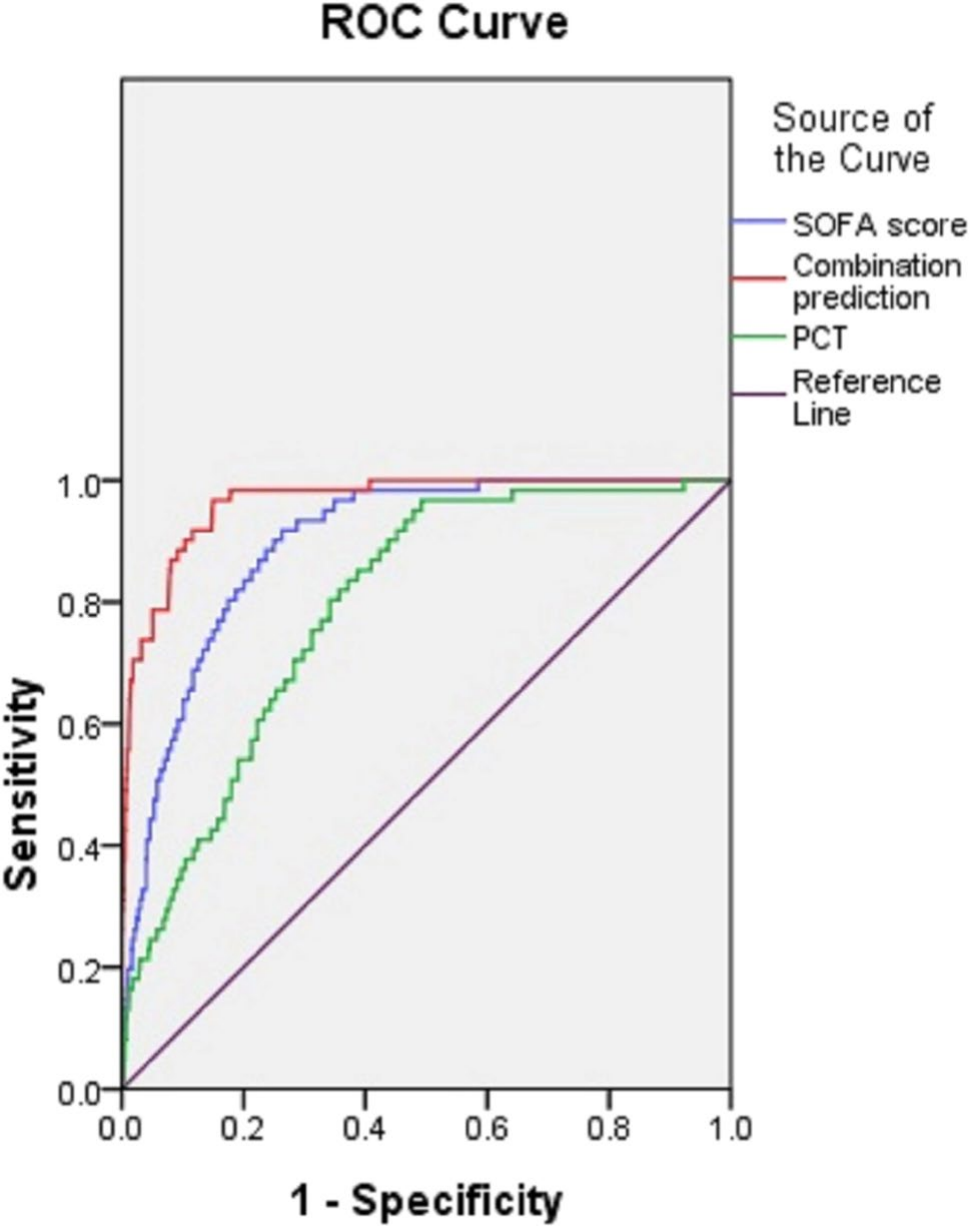
ROC curves of the SOFA score, PCT and their combination for prediction of septic shock after PCNL

The ROC curves (Fig. 1) demonstrated that the AUCs of SOFA score and PCT for predicting septic shock after PCNL were 0.896 (SE: 0.016, 95% CI 0.866–0.927) and 0.792 (SE: 0.024, 95% CI 0.744–0.839), respectively. The AUC of SOFA score was higher than that of PCT (*Z* = 3.606, *P* < 0.001). The optimal cut-off values were 4 and 11.56 ng/mL, respectively, for SOFA score and PCT. The ROC curve of their combination for predicting septic shock after PCNL was drawn using the probability obtained from logistic regression analysis, which had an AUC of 0.971 (SE 0.009, 95% CI 0.949–0.990). The predictive value of combination prediction of SOFA score and PCT was higher compared with individual prediction of SOFA score or PCT (AUC: 0.971 vs 0.896, *Z* = 4.086, *P* < 0.001; 0.971 vs 0.792, *Z* = 6.983, *P* < 0.001). Their clinical utility indexes for prediction of septic shock after PCNL were detailedly described in Table 3.

**Table 3:** Clinical utility indexes of SOFA score, PCT and their combination for predicting septic shock after PCNL

	Optimal cut-off	Sensitivity (%)	Specificity (%)	Accuracy (%)	Youden index
PCT (ng/mL)	11.56	80.3	68.8	69.4	0.49
SOFA score	4	93.4	75.4	76.2	0.69
Combination of SOFA score and PCT		98.4	86.7	87.2	0.85

*SOFA* sequential organ failure assessment, *PCT* procalcitonin

## Discussion

Sepsis is a complicated systemic disease characterized by a systemic inflammatory response. The original definition of sepsis was presence of systemic inflammatory response syndrome (SIRS) with suspected or verified infection (sepsis-1) [15]. Recently, the Sepsis-3 Task Force redefined sepsis based on the SOFA score that was proposed to substitute previous definition deriving from SIRS [4]. Several investigations have employed SOFA or quick SOFA (qSOFA) scores to predict adverse outcomes of sepsis patients, including mortality, ICU admission and organ dysfunction [16–18]. Additionally, Shi et al. evaluated the prognostic predictive values of SIRS, qSOFA and SOFA among patients with urolithiasis-related sepsis after surgical interventions, and recommended urologists to utilize SOFA score to predict prolonged ICU stay or in-hospital mortality among these patients according to evaluating results [19]. Peng et al. assessed the predictive values of SIRS, SOFA and qSOFA for septic shock in consecutive patients undergoing PCNL, and demonstrated slightly greater performance of SOFA in prediction of septic shock after PCNL compared with qSOFA and SIRS [11]. Shen et al. further investigated whether gender affect the performance of the SOFA score in prediction of septic shock after PCNL, and found that the performance of the gender-adjusted SOFA score in prediction of septic shock was comparable with the original SOFA score [20]. According to our results, the AUC of the SOFA score in prediction of septic shock after PCNL was 0.896 demonstrating a high predictive value, which was comparable with the previous reports.

On this basis, we added the serum PCT level to the SOFA score for prediction of septic shock after PCNL. As a nonspecific inflammation index, PCT is encoded by the calcitonin-I (CALC-1) gene. PCT possesses several advantages such as short induction time of bacterial stimulation, wide biological characteristics and a long half-life. Therefore, it has been widely applied in discriminating between infectious and non-infectious inflammation [21–25]. The serum PCT level is very low at a relative equilibrium in the body under normal physiological conditions. It increases rapidly following sepsis induced by an inflammatory stimulus associated with pathogen infection. PCT has been demonstrated to be a useful reference biomarker for sepsis, seriousness of sepsis, prognosis of sepsis and antibiotic management guidance [26–30]. Additionally, PCT can also be used to predict septic shock [31–33]. Song et al. demonstrated that the AUC of PCT for distinguishing septic shock from sepsis was 0.73 (95% CI 0.63–0.83, *P* < 0.001), and the AUC of PCT for distinguishing sepsis from the control group was 0.80 for PCT (95% CI 0.86–0.96, *P* < 0.001) [34]. Spoto et al. found that PCT could be applied in evaluating the evolution from sepsis toward septic shock, and PCT had a significant higher diagnostic ability to identify septic shock patients from septic patients compared with Mid-regional pro Adrenomedullin [35]. The meta-analysis on the predictive value of PCT for sepsis demonstrated that PCT was a useful laboratory indicator for early prediction of sepsis [12, 36], and PCT had higher specificity and accuracy compared with CRP [36]. Therefore, PCT was selected as a supplement of the SOFA score for prediction of septic shock after PCNL. Our results showed that PCT had a moderate predictive value with an AUC of 0.792, and addition of PCT to the SOFA score could elevate the predictive value for septic shock. In summary, both the SOFA score and PCT could be applied in predicting septic shock after PCNL, and their combination could further elevate the diagnostic ability.

This study had two main limitations, including a small sample size of septic shock patients and intrinsic limitations of retrospective studies. In the next step, we will further confirm the conclusion through a prospective study with a larger sample size.

## Conclusions

The SOFA score, PCT and operative time were independent risk factors of septic shock after PCNL. Both the SOFA score and PCT could be applied in prediction of septic shock after PCNL, and their combination could further elevate the diagnostic ability.

## Supplementary Information

Below is the link to the electronic supplementary material.

Supplementary file1 (XLS 507 KB)

## References

[1] Güler A, Erbin A, Ucpinar B (2019). Comparison of miniaturized percutaneous nephrolithotomy and standard percutaneous nephrolithotomy for the treatment of large kidney stones: a randomized prospective study. Urolithiasis.

[2] Liu J, Zhou C, Gao W (2020). Does preoperative urine culture still play a role in predicting postPCNL SIRS? A retrospective cohort study. Urolithiasis.

[3] Lipinska-Gediga M (2016). Sepsis and septic shock-is a microcirculation a main player. Anaesthesiol Intensive Ther.

[4] Singer M, Deutschman CS, Seymour CW (2016). The third international consensus definitions for sepsis and septic shock (Sepsis-3). JAMA.

[5] de la Rosette J, Assimos D, Desai M, CROES PCNL Study Group (2011). The clinical research office of the endourological society percutaneous nephrolithotomy global study: indications, complications, and outcomes in 5803 patients. J Endourol.

[6] Kreydin EI, Eisner BH (2013). Risk factors for sepsis after percutaneous renal stone surgery. Nat Rev Urol.

[7] Mirheydar HS, Palazzi KL, Derweesh IH (2013). Percutaneous nephrolithotomy use is increasing in the United States: an analysis of trends and complications. J Endourol.

[8] Chen D, Jiang C, Liang X (2019). Early and rapid prediction of postoperative infections following percutaneous nephrolithotomy in patients with complex kidney stones. BJU Int.

[9] Koras O, Bozkurt IH, Yonguc T (2015). Risk factors for postoperative infectious complications following percutaneous nephrolithotomy: a prospective clinical study. Urolithiasis.

[10] Prasad PA, Fang MC, Abe-Jones Y (2020). Time to recognition of sepsis in the emergency department using electronic health record data: a comparative analysis of systemic inflammatory response syndrome, sequential organ failure assessment, and quick sequential organ failure assessment. Crit Care Med.

[11] Peng Y, Zhang W, Xu Y (2021). Performance of SOFA, qSOFA and SIRS to predict septic shock after percutaneous nephrolithotomy. World J Urol.

[12] Wacker C, Prkno A, Brunkhorst FM (2013). Procalcitonin as a diagnostic marker for sepsis: a systematic review and meta-analysis. Lancet Infect Dis.

[13] Schuetz P, Christ-Crain M, Thomann R, ProHOSP Study Group (2009). Effect of procalcitonin-based guidelines versus standard guidelines on antibiotic use in lower respiratory tract infections: the ProHOSP randomized controlled trial. JAMA.

[14] Bouadma L, Luyt CE, Tubach F, PRORATA trial group (2010). Use of procalcitonin to reduce patients' exposure to antibiotics in intensive care units (PRORATA trial): a multicentre randomised controlled trial. Lancet.

[15] Bone RC, Balk RA, Cerra FB (1992). Definitions for sepsis and organ failure and guidelines for the use of innovative therapies in sepsis. The ACCP/SCCM consensus conference committee. American college of chest physicians/society of critical care medicine. Chest.

[16] Finkelsztein EJ, Jones DS, Ma KC (2017). Comparison of qSOFA and SIRS for predicting adverse outcomes of patients with suspicion of sepsis outside the intensive care unit. Crit Care.

[17] Pandey S, Sankhwar SN, Goel A (2019). Quick sequential (sepsis related) organ failure assessment: a high performance rapid prognostication tool in patients having acute pyelonephritis with upper urinary tract calculi. Investig Clin Urol.

[18] Yaghoubian A, Batter T, Mozafarpour S, Edge Research Consortium (2019). Use of the quick sequential organ failure assessment score for prediction of intensive care unit admission due to septic shock after percutaneous nephrolithotomy: a multicenter study. J Urol.

[19] Shi B, Shi F, Xu K (2019). The prognostic performance of sepsis-3 and SIRS criteria for patients with urolithiasis-associated sepsis transferred to ICU following surgical interventions. Exp Ther Med.

[20] Shen R, Zhang W, Ming S (2021). Gender-related differences in the performance of sequential organ failure assessment (SOFA) to predict septic shock after percutaneous nephrolithotomy. Urolithiasis.

[21] Angeletti S, Battistoni F, Fioravanti M (2013). Procalcitonin and mid-regional pro-adrenomedullin test combination in sepsis diagnosis. Clin Chem Lab Med.

[22] Angeletti S, Spoto S, Fogolari M (2015). Diagnostic and prognostic role of procalcitonin (PCT) and MR-pro- Adrenomedullin (MR-proADM) in bacterial infections. APMIS.

[23] Angeletti S, Dicuonzo G, Fioravanti M (2015). Procalcitonin, MR-proadrenomedullin, and cytokines measurement in sepsis diagnosis: advantages from test combination. Dis Markers.

[24] Angeletti S, Ciccozzi M, Fogolari M (2016). Procalcitonin and MR-proAdrenomedullin combined score in the diagnosis and prognosis of systemic and localized bacterial infections. J Infect.

[25] Spoto S, Cella E, de Cesaris M (2018). Procalcitonin and MR-Proadrenomedullin combination with SOFA and qSOFA scores for sepsis diagnosis and prognosis: a diagnostic algorithm. Shock.

[26] Moyer MW (2012). New biomarkers sought for improving sepsis management and care. Nat Med.

[27] Uzzan B, Cohen R, Nicolas P (2006). Procalcitonin as a diagnostic test for sepsis in critically ill adults and after surgery or trauma: a systematic review and meta-analysis. Crit Care Med.

[28] Gai L, Tong Y, Yan BQ (2018). Research on the diagnostic effect of PCT level in serum on patients with sepsis due to different pathogenic causes. Eur Rev Med Pharmacol Sci.

[29] Zhao JJ, Lou XL, Chen HW (2018). Diagnostic value of decoy receptor 3 combined with procalcitonin and soluble urokinase-type plasminogen activator receptor for sepsis. Cell Mol Biol Lett.

[30] Mustafić S, Brkić S, Prnjavorac B (2018). Diagnostic and prognostic value of procalcitonin in patients with sepsis. Med Glas (Zenica).

[31] Kade G, Literacki S, Rzeszotarska A (2018). Removal of procalcitonin and selected cytokines during continuous veno-venous hemodialysis using high cutoff hemofilters in patients with sepsis and acute kidney injury. Blood Purif.

[32] Pravin Charles MV, Kalaivani R, Venkatesh S (2018). Evaluation of procalcitonin as a diagnostic marker in neonatal sepsis. Indian J Pathol Microbiol.

[33] Wu CC, Lan HM, Han ST (2017). Comparison of diagnostic accuracy in sepsis between presepsin, procalcitonin, and C-reactive protein: a systematic review and meta-analysis. Ann Intensive Care.

[34] Song J, Park DW, Moon S (2019). Diagnostic and prognostic value of interleukin-6, pentraxin 3, and procalcitonin levels among sepsis and septic shock patients: a prospective controlled study according to the Sepsis-3 definitions. BMC Infect Dis.

[35] Spoto S, Fogolari M, De Florio L (2019). Procalcitonin and MR-proAdrenomedullin combination in the etiological diagnosis and prognosis of sepsis and septic shock. Microb Pathog.

[36] Tan M, Lu Y, Jiang H (2019). The diagnostic accuracy of procalcitonin and C-reactive protein for sepsis: a systematic review and meta-analysis. J Cell Biochem.

